# Diagnostics of Inherited Metabolic Diseases in Newborns with the Hyperammonemia Syndrome at the Onset of Disease (Pilot Study)

**DOI:** 10.17691/stm2021.13.1.07

**Published:** 2021-02-28

**Authors:** A.N. Kolchina, E.E. Yatsyshina, L.V. Malysheva, E.E. Ledentsova, E.E. Lidyaeva, O.V. Khaletskaya

**Affiliations:** PhD Student, Department of Hospital Pediatrics, Privolzhsky Research Medical University, 10/1 Minin and Pozharsky Square, Nizhny Novgorod, 603005, Russia; Associate Professor, Department of Hospital Pediatrics, Privolzhsky Research Medical University, 10/1 Minin and Pozharsky Square, Nizhny Novgorod, 603005, Russia; Head of the Clinical Diagnostic Laboratory, Children’s City Clinical Hospital No.1, 76 Prospect Gagarina, Nizhny Novgorod, 603081, Russia; Physician of Clinical Laboratory Diagnostics, Children’s City Clinical Hospital No.1, 76 Prospect Gagarina, Nizhny Novgorod, 603081, Russia; Anesthesiologist-Resuscitator, Resuscitation and Intensive Care Unit, Children’s City Clinical Hospital No.1, 76 Prospect Gagarina, Nizhny Novgorod, 603081, Russia; Professor, Head of the Department of Hospital Pediatrics, Privolzhsky Research Medical University, 10/1 Minin and Pozharsky Square, Nizhny Novgorod, 603005, Russia

**Keywords:** hyperammonemia syndrome, metabolic disease in newborns, neonatal diagnosis, inherited metabolic diseases, aciduria

## Abstract

**The aim of the study** was to develop a diagnostic model that allows with a high degree of probability predicting the development of inherited metabolic disease (IMD) in newborns with the hyperammonemia syndrome at the onset of disease and determine the adequate management tactics for such patients.

**Materials and Methods.:**

The study included 15 female and 5 male infants with the hyperammonemia syndrome. All the patients underwent a full range of clinical laboratory studies in accordance with the standards of treatment of the underlying disease. Blood ammonia measuring (quantitative assessment) was carried out on the basis of the biochemical laboratory of the Children’s City Clinical Hospital No.1 (Nizhny Novgorod, Russia) using a portable PocketChem BA blood ammonia meter (Japan) and Ammonia Test Kit II test strips (Japan) in accordance with the manufacturer’s protocol. The confirmation of the IMD diagnosis was carried out using the tandem mass spectrometry (TMS) method and molecular genetic testing (genome sequencing).

To check the assumptions, all the patients during the follow-up were randomized retrospectively into two groups depending on the TMS results and molecular genetic testing. Group 1 (n=8) included the patients diagnosed with IMD and group 2 (n=12) included the patients with transient hyperammonemia in the neonatal period.

**Results.:**

The clinical manifestations of the hyperammonemia syndrome in the neonatal period are most frequently realized in the form of a syndrome of CNS depression of varying degrees up to coma (75%), a convulsive syndrome (55%), vomiting (40%) without significant differences in the frequency of occurrence due to the causes of hyperammonemia. In a third of cases, the onset of the hyperammonemia syndrome occurs in the early neonatal period.

The ammonia levels in patients with hyperammonemia caused by IMD are statistically significantly higher than those in patients with transient hyperammonemia (p=0.014) which may serve as the first diagnostic sign of IMD in a newborn.

A tendency to a more frequent development of anemia in infants with IMD (p=0.084) has been established which might be due to the compensation of metabolic acidosis. The presence of anemia in combination with clinical features and the level of hyperammonemia increases the risk of IMD.

In the course of the study, a diagnostic model was developed for predicting the risk of IMD development in a newborn at the onset of hyperammonemia. Through the discriminant analysis, a statistically significant relationship was established between the level of ammonia at the onset, blood glucose and base deficiency levels, blood lactate, hemoglobin, erythrocyte levels, the average volume of erythrocytes and the pH level (p=0.040) with the risk of IMD development. The sensitivity of the model has amounted to 87.5%, the specificity — 83.3%. The diagnostic efficiency of the model is 85.0%.

**Conclusion.:**

The proposed model can be used at detecting the debut of the hyperammonemia syndrome in newborns in order to predict the probability of IMD and work out the adequate tactics of the management for these patients.

## Introduction

An excessive amount of ammonia in the body resulted from improperly working catabolic processes presents a serious biochemical problem. This is due to the toxicity of ammonia and, primarily, its effect on the central nervous system [1, 2]. Hyperammonemia is a condition in which the level of ammonia is above 100 μmol/L in newborns and above 50 μmol/L in adults [3]. It accompanies a wide range of pathologies. First of all, these are inherited metabolic diseases (IMD), which can occur at different ages, the diseases with their manifestation in the neonatal period having the most severe consequences. In this case, during the neonatal period, hyperammonemia should be differentiated from transient hyperammonemia of newborns, which is associated with insufficient maturity of the enzymatic systems of the liver. In particular, it is due to this that the normal level of blood ammonia in a newborn is slightly higher than that in infants over 1 month. This condition develops as a result of the activation of catabolic processes that provide conditions for gluconeogenesis, the redistribution of nutrients and energy between organs with their predominant delivery to those that play a crucial role in the adaptation of a newborn to the new environment. However, the factors that complicate the normal course of the neonatal period may induce pathological hyperammonemia. First of all, hypoxic lesions can be refered to these factors. Hypoxic stress enhances protein catabolism in the newborn’s body, which leads to a sharp increase in the blood ammonia level [4]. Secondary hyperammonemia is associated with liver damage or taking medications such as valproic acid preparations [5].

An increase in the blood ammonia concentration leads to an increase in the ammonia level in the brain [6], which ultimately causes astrocyte swelling, an increase in the permeability of the blood-brain barrier, changes in cerebral metabolism and neurotransmission, as well as cerebral edema [7, 8]. Ammonia also interferes with potassium buffering in astrocytes. The resulting increase in the extracellular potassium concentration and hyperactivation of the neuronal Na^+^-K^+^-2Cl-cotransporter worsen cortical inhibitory neuromediation and induce neurological dysfunction that leads to seizures [8, 9].

The brain’s protective barrier against high ammonia levels is the glutamine synthetase activity in astrocytes. However, this detoxification system is limited by osmotic stress, which is induced by the glutamine accumulation in astrocytes (the so-called ammonia–glutamine–brain edema hypothesis) [10]. All these mechanisms determine the main symptoms in the clinical picture of newborns with the hyperammonemia syndrome such as depression of consciousness of various levels, seizures, vomiting, or frequent regurgitation. The appearance of these symptoms in the clinic is observed when the ammonia level reaches 300 μmol/L [11]. According to other authors, the manifestation is noted at higher values of ammonia — 400–500 μmol/L [12].

In the neonatal period, the differential diagnosis of the causes of hyperammonemia, and, primarily IMD and transient hyperammonemia of newborns, is very important, since it requires taking quick decisions on the choice of further treatment of patients. However, neonatal screening, firstly, does not cover all nosologies accompanied by hyperammonemia, and, secondly, the manifestation of severe disorders of the urea cycle occurs on day 2–3 of a newborn’s life, when the neonatal screening has not even been performed yet. Tandem mass spectrometry (TMS) and molecular genetic testing (with a good family history) also take time [13]. Therefore, a serious toxic effect of hyperammonemia on the central nervous system and high risk of disability and lethal outcome cause the necessity and importance of quick diagnosing of this condition to start therapy [14]. In this regard, the search for new differential diagnostic approaches to determine the tactics of managing patients with the hyperammonemia syndrome at the onset of the disease has become **the aim of our study.**

The task was to develop a diagnostic model that allows predicting with high probability the development of IMD in newborns with the hyperammonemia syndrome at the onset of the disease and choosing the optimal treatment tactics in order to reduce unfavorable outcomes.

## Materials and Methods

In the period from 2017 to 2020, 20 full-term infants with the hyperammonemia syndrome were examined at the department of full-term and premature infants and the resuscitation and intensive care unit of the multidisciplinary children’s hospital “Children’s City Clinical Hospital No.1” in Nizhny Novgorod (Russia). The study was carried out in accordance with the Declaration of Helsinki (2013) and approved by the Ethics Committee of the Privolzhsky Research Medical University.

The study included 15 female babies (71.4±12.1%) and 5 male babies (28.6±0.7%). The inclusion criteria were a signed informed consent of the patients’ parents to participate in the study in accordance with Federal Law No.323 of November 21, 2011 “On the Foundations of Health Protection in the Russian Federation” and the presence of the hyperammonemia syndrome.

All the newborns underwent a full range of clinical laboratory studies in accordance with the standards of treatment of the underlying disease. Blood ammonia measuring (quantitative assessment) was performed in the biochemical laboratory of the Children’s City Clinical Hospital No.1 using a portable PocketChem BA blood ammonia meter and Ammonia Test Kit II test strips (Japan) in accordance with the manufacturer’s protocol. The confirmation of the IMD diagnosis was carried out using the TMS method and molecular genetic testing (genome sequencing). The rest of the laboratory studies were performed according to the indications to confirm the nosological form of IMD.

To check the assumptions, all the patients during the follow-up were randomized retrospectively into two groups depending on the results of TMS and molecular genetic testing. Group 1 (n=8) included the patients with an established diagnosis of IMD and group 2 (n=12) consisted of the patients with transient hyperammonemia in the neonatal period. Further comparison of clinical and laboratory features was carried out within the indicated groups.

In group 1, with IMD revealed by the results of TMS, genome sequencing was performed in 4 cases and the following nosological forms of IMD were confirmed: 2 cases of methylmalonic aciduria, 1 case of non-ketotic hyperglycemia, 1 case of urea cycle disorder (ornithine transcarbamylase deficiency). The other 4 patients demonstrated the changes characteristic of IMD according to the TMS data; two of them did not undergo genome sequencing due to their death until the results of TMS were obtained. Two sequencing results are in progress at the time of publication. In this regard, these cases at the time of the study were regarded as unspecified IMD. The time frame for TMS was 14 days, for molecular genetic research — 90 days.

### Statistical data processing.

Statistical data are presented as Me [Q1; Q3]. The results were processed using the IBM SPSS Statistics program. The parameters were interpreted using the Mann–Whitney test as a nonparametric test for comparing two independent samples. Fisher’s exact test was used for the analysis of the nominal data, the method of discriminant analysis with forced inclusion of the studied factors was used to develop the predictive model.

## Results and Discussion

At increased levels of blood ammonia in newborns, direct toxic damage to the central nervous system is observed; it is neurological disorders in the neonatal period that serve as the main criterion for the indication for ammonia measuring. The diagnosis of hyperammonemia in the newborns was carried out provided the child had a syndrome of CNS depression of varying severity, seizures, frequent regurgitation, or vomiting, which could not be explained by organic causes.

According to the data obtained, hyperammonemia was most often accompanied by a syndrome of CNS depression of varying severity up to coma (75%), a convulsive syndrome (55%), and vomiting (40%).

The syndrome of CNS depression in our case was noted in 15 cases (75.0%), 8 cases of them (66.7%) had transient hyperammonemia and 7 cases (87.5%) had IMD. A convulsive syndrome was detected in 11 patients (55%), 5 infants of those were subsequently diagnosed with IMD and 6 patients had transient hyperammonemia. Vomiting was noted in 8 cases (40%), 4 of them (50%) were the patients with IMD.

The onset of the clinical manifestations in the first week of life (in the early neonatal period) was observed in 5 out of 20 patients. Thus, a quarter of patients manifested the hyperammonemia syndrome in the early neonatal period. The debut of hyperammonemia in the remaining 15 cases corresponded to the late neonatal period (14 [9; 29] days).

The patients diagnosed with IMD were characterized by a progressive course of the disease with subsequent development of persistent neurological deficit. On the other hand, the patients with severe transient hyperammonemia (ammonia levels above 200 μmol/L) also had pronounced CNS disorders and incomplete regression of symptoms thereafter. This fact significantly complicates early differential diagnosis and determination of patient management tactics.

Comparing the frequency of clinical manifestations in the groups of the patients with transient hyperammonemia and IMD by performing Fisher’s exact test for comparing the nominal data, no statistically significant differences between the two groups were found, which also indicates the impossibility of a clear differential diagnosis of IMD and transient hyperammonemia based only on clinical manifestations. Thereby, there arises a need to search and develop a new diagnostic approach that allows one to assume the presence or absence of IMD in a patient in the absence of TMS data and molecular genetic testing in the first days of the onset of the disease.

When comparing the ammonia levels depending on the presence or absence of IMD with the use of the Mann–Whitney test, statistically significant differences in its values were revealed in the patients with IMD and those with transient hyperammonemia (p=0.014). In the presence of IMD, the ammonia levels were significantly higher (179.20 [146.05; 219.15] μmol/L) than in the group with transient hyperammonemia (119.90 [101.70; 189.25] μmol/L). The results are presented in the Figure.

In the course of the study, in these groups, the general clinical and biochemical blood counts were also compared depending on the presence or absence of IMD. The analysis of the laboratory findings revealed a tendency towards the development of anemia in the patients of group 1 with IMD. The level of significance of the differences by the frequency of anemia in the patients of the compared groups was close to the statistically significant one (p=0.084). A decrease in the hemoglobin level was observed in half of the subjects, 6 cases with IMD and 1 case with transient hyperammonemia among them. At the same time, no evidence of a toxic effect of ammonia on the hematopoietic system was found in the available literature, which might lead to the development of anemia. The hemoglobin buffer system is more likely to be involved in the development of anemia [15].

**Figure:**
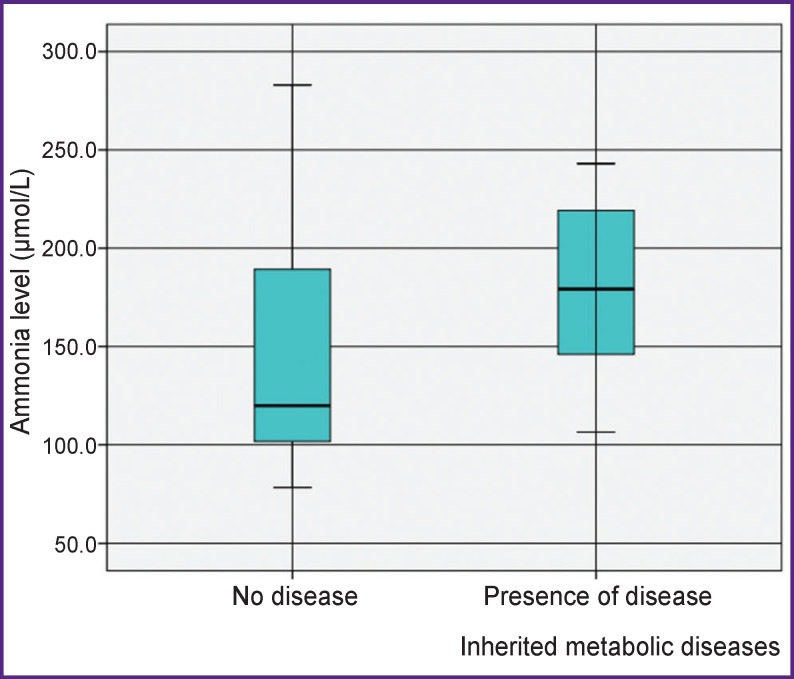
Distribution of values of the ammonia levels in newborns depending on the presence and absence of inherited metabolic disease

**Table:** Characteristics of the model for determining the presence of inherited metabolic disease in newborns obtained by the discriminant analysis

IMD	Predicted affiliation to the group		Total
	No disease	Presence of disease	
No (n)	10 (TN)	2 (FP)	12
Yes (n)	1 (FN)	7 (TP)	8
No (%)	87.5 (TN)	12.5 (FP)	100.0
Yes (%)	16.7 (FN)	83.3 (TP)	100.0

Here: TN — a true negative result; TP — true positive; FN — false negative; FP — false positive.

The other laboratory findings did not have statistically significant differences in their values between the groups.

Taking into account all the complexities of the differential diagnosis of the hyperammonemia syndrome, as well as the duration of the performance of clarifying tests, a diagnostic model for predicting the risk of developing IMD in a newborn with the hyperammonemia syndrome was developed during the study. Considering the statistically significant differences between the values of the ammonia levels in the patients of the compared groups, as well as probable significance of other factors in the diagnosis of IMD, we assessed the effect of the combination of factors by the method of discriminant analysis with forced inclusion of the studied factors. During the analysis, the factors that have the greatest effect on the level of statistical significance of the mathematical model were identified.

To determine the risk of IMD development, the following equation was proposed:

$$Y_{IMD}=-104.371-0.016X_{NH_{4}}++0.273X_{LACTATE}+0.566X_{GLU}+0.001X_{Hb}++0.882X_{RBC}+0.044X_{MCV}-0.108X_{BE}+12.953X_{pH,}$$

where *Y_IMD_* is the discriminant function characterizing the probability of IMD presence; *X_NH4_* — an ammonia value (μmol/L); *X_LACTATE_* — blood lactate (mmol/L); *X_GLU_* — blood glucose (mmol/L); *X_Hb_* — hemoglobin (g/L); *X_RBC_* — erythrocytes (×10^12^/L); *X_MCV_* — the average volume of erythrocytes (fL); *X_BE_* — base excess (IU); *X_pH_* — blood pH.

The constant of discrimination dividing the subjects into two groups was defined as the value of the function equidistant from the centroids, which were 0.852 in the group with no IMD and –1.136 in the group with IMD. The discrimination constant is –0.142.

The statistically significant differences were determined (p=0.040) at comparing the mean values of the discriminant function in both groups using the Wilks’ λ coefficient.

The patients’ affiliation to the group of either high or low risk of IMD development was determined on the basis of the calculated values of the predictive discriminant function. With the value of *Y_IMD_* above –0.142, the patient was referred to the group of high risk of IMD, with the value of *Y_IMD_* below –0.142, the patient was referred to the group of low risk of IMD.

The model’s sensitivity was 87.5% (7 correctly predicted cases with IMD out of 8), specificity — 83.3% (10 correctly predicted cases with no IMD out of 12). The diagnostic efficiency of the model was 85.0% (17 correctly predicted cases with IMD and no IMD in the studied population). The characteristics of the developed model are presented in the Table.

In order to illustrate the applicability of this model in practice, consider the following clinical examples.

### Clinical case 1.

*A female patient M., born from her mother’s 1^st^ pregnancy (Gravida 1, Para 1), against the background of mild anemia. The mother gave birth via a normal vaginal delivery at 40–41 weeks’ gestation, with the baby’s cephalic presentation.*

*Amniotomy was performed, the amniotic fluid was clear. The Apgar scores were 8/8. Birth weight was 3900 g. On day 2 of life, the baby’s condition deteriorated sharply, there was weakness, regurgitation with infant formula, a significant decrease in body weight, by 540 g, was noted. On day 3, the condition was assessed as very severe, there was a pronounced depression of consciousness (weakness, areflexia, atony), convulsive readiness. On day 4, the infant was transferred to the ICU of a multidisciplinary clinical hospital.*

*The leading syndromes in the clinical picture were the 1^st^ stage of coma, neonatal seizures. The stigmas of dysembryogenesis revealed were hypertelorism, elongated filter, dysotia, short neck, flat forehead, high arch of the hard palate.*

*The laboratory findings showed: the level of ammonia — 165.0 μmol/L; lactate — 5.1 mmol/L; glucose — 21 mmol/L; Hb — 118 g/L; RBC — 3.69·1012/L; MCV — 67 fL; BE — 5.0; pH — 7.4. We enter the data into our formula:*

−104.371−0.016⋅165.0+0.273⋅5.1++0.566⋅21.0+0.001⋅118.0+0.882⋅3.69++0.044⋅67.0−0.108⋅(−5.0)++12.953⋅7.405=9.045.

*The Y_IMD_ value is higher than –0.142, therefore, the patient belongs to the group of high risk of IMD.*

*During TMS, a sharp increase in the level of propionyl-L-carnitine was found, the urine analysis for organic acids showed an elevated level of methylmalonic acid — up to 5650 mmol/L. The genetic research revealed a change in the nucleotide sequence c.1847G>C leading to its replacement by p.Arg616Pro in a homozygous state, not described in the database on mutations and polymorphisms. The presence of this mutation in the infant, an increase in the level of methylmalonic acid according to the urine analysis for organic acids, an increase in the level of propionyl-L-carnitine according to the TMS data confirms the presence of such IMD in this patient as methylmalonic aciduria. In the mother of the proband, the p.Arg616Pro mutation was detected in a heterozygous state.*

This example illustrates a possibility of predicting the risk of developing IMD in the patient.

### Clinical case 2.

*A female patient R. was admitted to the department of pathology of newborns and premature babies with a severe central nervous system depression syndrome, neonatal convulsions on day 3 of life. She was born from her mother’s 2^nd^ pregnancy (Gravida 2, Para 2) via a 2 urgent normal vaginal delivery, with cephalic presentation. Labor weakness was observed, oxytocin stimulation was performed. The Apgar scores were 8/8, birth weight — 3120 g. The leading syndromes were a syndrome of central nervous system depression (sopor), neonatal convulsions. The laboratory findings showed: the ammonia level — 137.8 μmol/L; lactate — 1.1 mmol/L; glucose — 3.60 mmol/L; Hb — 150 g/L; RBC — 5.80·1012/L; MCV — 95.7 fL; BE — 7.2; pH — 7.2. We enter the data into the formula:*

−104.371−0.016⋅137.8+0.273⋅1.1++0.566⋅3.60+0.001⋅150.0+0.882⋅5.80++0.044⋅95.7−108⋅(−7.2)+12.953⋅7.243=−0.165.

*The Y_IMD_ value is below –0.142, therefore, the patient belongs to the group of low risk for the development of IMD.*

*According to TMS and the urine analysis for organic acids, no signs of IMD were revealed.*

Thus, the clinical manifestations of the hyperammonemia syndrome in the neonatal period are most often realized as a syndrome of central nervous system depression up to coma (75%), a convulsive syndrome (55%), vomiting (40%) without significant differences in the frequency of occurrence depending on the causes of hyperammonemia. A quarter of cases of the debut of the hyperammonemia syndrome occur in the early neonatal period.

The ammonia level in patients with hyperammonemia due to IMD is statistically significantly higher than in patients with transient hyperammonemia (p=0.014), which may serve as the first diagnostic sign of IMD presence in a newborn.

A tendency towards a more frequent development of anemia in patients with IMD (p=0.084) was established, which may occur due to the compensation of metabolic acidosis. The presence of anemia in combination with clinical features and an elevated level of hyperammonemia increases the risk of IMD in patients.

## Conclusion

In the course of the study, a diagnostic model for predicting the risk of developing inherited metabolic disease in a newborn at the onset of hyperammonemia was developed. The sensitivity of the model was 87.5%, specificity — 83.3%, diagnostic efficiency — 85.0%. The proposed model can be used at the beginning of detecting the hyperammonemia syndrome in newborns in order to predict the probability of detecting inherited metabolic disease in these infants and develop adequate management tactics.

It will make it possible to diagnose inherited metabolic diseases at an early stage before the results of molecular genetic testing are obtained. This allows identifying a high-risk group for the development of these diseases and correcting treatment tactics in time.
